# Transmittance and Reflectance Effects during Thermal Diffusivity Measurements of GNP Samples with the Flash Method

**DOI:** 10.3390/ma12050696

**Published:** 2019-02-27

**Authors:** Stefano Bellucci, Gianluigi Bovesecchi, Antonino Cataldo, Paolo Coppa, Sandra Corasaniti, Michele Potenza

**Affiliations:** 1 INFN-Laboratori Nazionali di Frascati, Frascati, 00044 Rome, Italy; Stefano.Bellucci@lnf.infn.it (S.B.); antonino.e.cataldo@gmail.com (A.C.); 2 Department of Industrial Engineering, University of Rome “Tor Vergata”, 00133 Rome, Italy; coppa@uniroma2.it (P.C.); sandra.corasaniti@uniroma2.it (S.C.); m.potenza23@gmail.com (M.P.); 3 Neuroscience, Imaging and Clinical Science Department, University “G. d’Annunzio”, 66100 Chieti (Pescara), Italy

**Keywords:** transmittance, flash method, thermal diffusivity, graphene nano-platelets, extinction coefficient

## Abstract

Thermal diffusivity of *GNPs* (graphene nano-platelets) is an important thermo-physical property as it is useful to predict the material behavior in many heat transfer applications. *GNP* samples were pressed at different loads to obtain different densities, and then thermal diffusivity was measured with the flash method. All samples were coated with a thin layer (~1 µm) of colloidal graphite (Aquadag^®^) on both sides to reduce reflectance of their surfaces and consequently increase the emissivity. Carrying out measurements on both samples with and without coating, a difference between the two series of measurements was found: This is attributed to a non-negligible transmittance of the uncoated samples due to the porosity of *GNPs*. Furthermore, assuming a spatial distribution of the light within the samples according to the Lambert-Bougert-Beer law, the extinction coefficient of *GNP* at different densities has been evaluated processing experimental data with a nonlinear least square regression, (*NL-LSF*, nonlinear least square fitting), whose model contains the extinction coefficient as unknown. The proposed method represents a further improvement of thermal diffusivity data processing, crucial to calculate the extinction coefficient when data with and without coating are available; or to correct biased thermal diffusivity data when the extinction coefficient is already known. Moreover, reflectance effects have been highlighted comparing asymptotic temperature reached during the tests on coated and uncoated samples at different densities. In fact, the decrease of asymptotic temperature of the uncoated samples gives the percentage of the light reflected and consequently an estimate of the reflectance of the *GNP* surface.

## 1. Introduction

Graphene, since its recent discovery, has attracted a growing attention in the scientific community due to its particular properties, e.g., [1]. There are different methods to produce graphene. Even if chemical vapor deposition is the most commonly used [2,3], an easier and quicker method is the exfoliation assisted by microwave irradiation: This procedure produces few layered graphene with micron sized lateral dimensions, called graphene nano-platelets (*GNP*) [4,5,6].

In the present work graphene nano-platelets in the form of free standing low density conglomerates were investigated.

Among the different peculiar properties of *GNP*, the thermo-physical ones result interesting, due to their influence on heat storage and propagation. Due to recent discovery of graphene, many data are still missing.

Thermal diffusivity is the thermo-physical property influencing the thermal behavior during transient evolution of materials. Furthermore, its determination is useful to evaluate thermal conductivity, when not possible or hard with other methods. Among the different methods to measure it, flash method [7] is perhaps the most commonly used due to its easy of application. Homemade systems can be easily assembled, and commercial instruments are also available (e.g., [8]). The procedure consists of irradiating a surface of a sample, in the shape of a thin slab, and measuring the temperature increase on the opposite side, preferably with a non-contact thermometer as a pyrometer. The method is particularly suited to slabs, even thin foils, and requires a pulse thermal source, generally a photographic flash or a laser.

But attention must be paid to some problems which can arise during measurements or data processing:

- a very short energy pulse can produce a meaningful temperature increase on the irradiated surface which can even damage it; this can be avoided with a low power source (a photographic flash), but temperature increase on the opposite side results very low, and temperature detection becomes difficult;
- an error can arise when the time length of the flash pulse is comparable with the temperature transition on the opposite face: In such case the approximation of the analytical trend of the pulse with a Dirac-delta function cannot be considered any more valid, and a meaningful systematic error is introduced [6,9].

**Figure 1:**
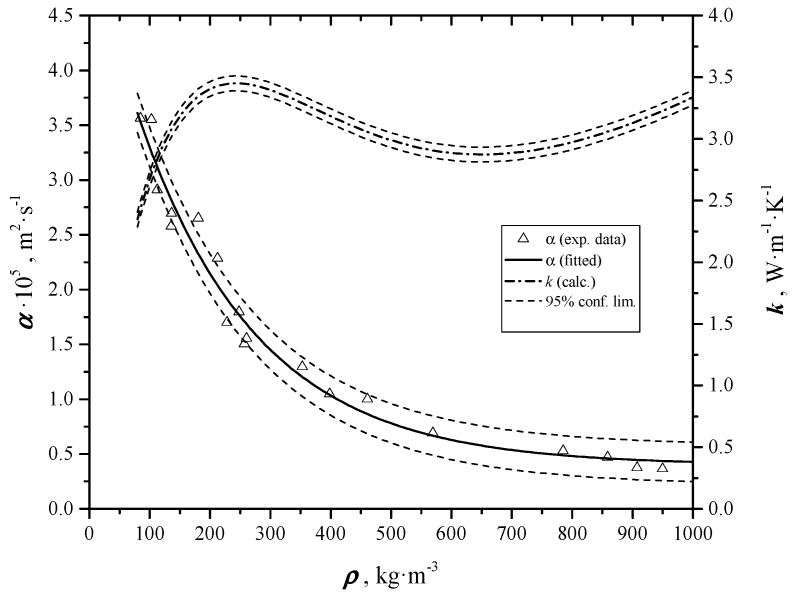
Trend of thermal diffusivity *α* and thermal conductivity *λ* as a function of density (*ρ*) [6].

In [6] the authors reported the evaluated thermal diffusivity data of graphene nano-platelets, in the shape of thin and flat disks, through a homemade flash method. Samples had been coated before measurements with a thin layer (~1μm of colloidal graphite, Aquadag^®^, by Agar Scientific Ltd, Stansted, Essex, UK) in order to make them opaque and good absorber in the infrared wavelength band used for measurements. The results are summarized in Figure 1. In order to avoid the error arising from the finite time length of the pulse, a special analytical solution was developed, which uses a suited regression analytical model in the nonlinear least square fitting of the experimental data.

**Figure 2:**
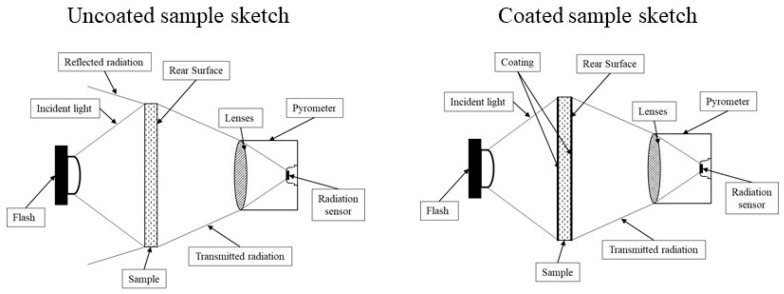
Sketches of the experiments.

Samples measured and reported in [6], see Figure 2, had been blackened with a thin layer of colloidal graphite (Aquadag^®^) in order to increase the emissivity of their surface. A higher absorbance of the irradiated surface is so obtained, producing an increase of the detector output. The coating thickness is so thin (~1 μm) to negligibly influence the obtained results.

When the samples were measured uncoated, values of thermal diffusivity lower than the coated ones were found. This difference results higher at lower densities, while at higher densities the two series of data practically coincide. The only possible explanation of this is a non-complete opacity of the uncoated samples: In fact, if the thermal wave impinging on the sample surface is not completely absorbed by it but penetrates in a layer of the sample due to its partial transparency, the transmitted heat arrives in advance to the rear surface, and thermal diffusivity results apparently higher.

Other authors dealt with the same or similar problems, e.g., [10,11]. The effect is much more evident when highly transparent material is tested (e.g., glasses [12,13]).

In the present paper, a different solution of the basic heat conduction equation is obtained in order to avoid this error. This solution is used for updating the *NL-LSF* model. The new model takes into account not only the time trend of the flash pulse but also the space distribution of the radiation into the samples, evaluated from the Lambert-Bougert-Beer law. This trend replaces the Dirac delta function of the thermal power absorbed by the irradiated surface in the case of completely opaque samples. The extinction coefficient behaves as an unknown parameter in the *NL-LSF* of experimental data, and its best estimate is supplied by the regression procedure.

The temperature asymptotic increase during tests was evaluated as explained in Section 5.2. Theoretically, being the supplied thermal power constant, this asymptotic temperature should not change when density increases, because mass and specific heat remain practically the same, also after compression. However, measurements carried out on the uncoated samples showed a linear decrease of the asymptotic temperature. This effect can be attributed to reflectance: In fact, when *GNP* samples are pressed with higher loads, their surfaces appear much smoother and shinier, i.e., their reflectance increases. Thus, even with the same supplied thermal power, the asymptotic temperature results smaller.

On the basis of the above mentioned considerations both the data of uncoated and coated samples were processed with two different regression models, the first taking into account the transmittance, while the second not. From the difference between the two results an estimation of the partial transmittance coefficient of the GNP uncoated samples is obtained. The main result of this procedure is twice: Either it is possible to evaluate the extinction coefficient of the graphene nanoplatelets when data of coated and uncoated samples are both measured, or row data of uncoated samples can be corrected through the use of the extinction coefficient already known or measured.

## 2. Analytical Solution

### 2.1. Parker Solution

Under the following hypotheses:

- homogeneous and isotropic materials;
- pulse heating (Dirac *δ*);
- adiabatic condition of the slab after the pulse;
- homogeneously irradiated surface;
- one-dimensional heat propagation;
- thermo-physical properties constant in the temperature range of the test;

the Parker solution [7] is used as regression model in *NL-LSF*. This procedure gives the best estimate of two quantities: The temperature increase (*T*_∞_ − *T*_0_) and the thermal diffusivity *α*.

(1) $$T(x,\tau )=T_{0}+\left(T_{\infty }-T_{0}\right)\left[1+2\sum_{n=1}^{\infty }\cos\left(\frac{n\pi x}{L}\right)\exp\left(-\frac{n^{2}\pi^{2}\alpha \tau }{L^{2}}\right)\right]$$

### 2.2. Double Exponential

When the time length of the flash pulse is comparable with the temperature transition on the opposite face, the hypothesis of pulse heating (Dirac *δ*) is no more applicable and consequently Equation (1) is no more suited for data processing. In literature [14,15] analytical solutions for square wave and triangular wave thermal pulse are also present.

In [6] a better description of the real flash signal was adopted, approximating the pulse with two overlapping exponentials, each with its own time constant:

(2) $$S(\tau )=A\left[\exp(-R_{1}\tau )-\exp(-R_{2}\tau )\right]$$

where *A* represents the signal intensity connected with the supplied energy, while *R*_1_, and *R*_2_ are the inverses of the two time constants, the first linked to the lamp filament temperature increase, and the second to the time constant of the flash capacitor discharge. The way to determine *R*_1_ and *R*_2_ is described in [6].

Using Equation (2) as input, in the way described by the Green function method [16], the solution is as follows (see Appendix A of [6]):

(3) $$\begin{array}{l} T(L,\tau )=A^{\prime }\left\{ \begin{array}{l} \frac{1-\exp\left(-R_{1}\tau \right)}{R_{1}}-\frac{\exp\left(-R_{2}\tau \right)-1}{R_{2}}+2\sum_{n=1}^{\infty }[{\left(-1\right)}^{n} \end{array}\right.\\ \times \left(\frac{\exp\left(R_{1}\tau \right)-\exp\left(-{\left(\frac{n\pi }{L}\right)}^{2}\alpha \tau \right)}{{\left(\frac{n\pi }{L}\right)}^{2}\alpha -R_{1}}-\frac{\exp\left(R_{2}\tau \right)-\exp\left(-{\left(\frac{n\pi }{L}\right)}^{2}\alpha \tau \right)}{{\left(\frac{n\pi }{L}\right)}^{2}\alpha -R_{2}}\right)]\} \end{array}$$

Equation (3) represents the temperature response of the rear surface (*x* = *L*) of a sample irradiated at *x* = 0 by a flash whose signal is described by Equations (2) and (3) has been used as regression model to fit the data both of coated and uncoated samples.

### 2.3. Solution Involving GNP Partial Transparency

*GNP* samples are soft and porous, and consequently the irradiated surfaces result no more perfectly opaque, but partially transparent. Equation (3) does not consider the samples transparency, and if it is used in data processing for uncoated *GNP* leads to an overestimation of the thermal diffusivity. To avoid this error some practical solutions must be adopted to increase absorbance of the irradiated surface, as for example coating the surfaces with a high absorbing medium [6]. Alternatively, an analytical model that takes into account the transmittance effects could be used in data processing. The previous section reports the temperature response of a sample when the thermal input pulse is a function of time described in Equation (2). For this purpose, the Green function method has been used, which allows to determine the temperature response of the sample for any type of pulse when the temperature response to a Dirac *δ* input is known. An analogue procedure can be followed when the thermal pulse is a function of both space and time. In first approximation, it is possible to assume a spatial distribution of the light penetrating into the sample as described by the Bougher-Beer law:

(4) $$I(x)=I_{0}\exp(-a_{\lambda }x)$$

where *I*_0_ is the light intensity in *x* = 0 and *a*_λ_ is the extinction coefficient, dependent on radiation wavelength and material. In a first step, *a*_λ_ can be assumed independent on wavelength, and the flash signal described by:

(5) $$S(\tau ,x)=B\cdot a\cdot \exp(-a\cdot x)\left[\exp(-R_{1}\tau )-\exp(-R_{2}\tau )\right]$$

Beside the inverse of two time constants of the flash *R*_1_ and *R*_2_, *B* is a factor proportional to the light intensity and *a* the extinction coefficient averaged in the wavelength range of the impinging light. Using Equation (5) as thermal input, the solution of the heat conduction equation is (details are reported in Appendix A):

(6) $$T(L,\tau )=B^{\prime }\left\{F(\tau )\left(1-\exp(-a\;L)\right)+2\sum_{n=1}^{\infty }\left[G(\tau ){\left(1+{\left(\frac{n\pi }{a\;L}\right)}^{2}\right)}^{-1}\left(-\exp(-a\;L)+{\left(-1\right)}^{n}\right)\right]\right\}$$

where,

$$F(\tau )=\frac{1-\exp\left(-R_{1}\tau \right)}{R_{1}}-\frac{\exp\left(-R_{2}\tau \right)-1}{R_{2}}$$

and,

$$G(\tau )=\frac{\exp\left(-R_{1}\tau \right)-\exp\left(-{\left(\frac{n\pi }{L}\right)}^{2}\alpha \tau \right)}{{\left(\frac{n\pi }{L}\right)}^{2}\alpha -R_{1}}-\frac{\exp\left(-R_{2}\tau \right)-\exp\left(-{\left(\frac{n\pi }{L}\right)}^{2}\alpha \tau \right)}{{\left(\frac{n\pi }{L}\right)}^{2}\alpha -R_{2}}$$

Equation (6) represents the temperature response in *x* = *L* when the sample is irradiated in *x* = 0 by a thermal pulse described by Equations (5) and (6) can be used in different ways in data processing. When the thermal diffusivity of the perfectly opaque material is known (e.g., of *GNP* coated samples with the standard procedure), processing data of uncoated samples using Equation (6) as regression model in *NL-LSF* gives the joint best estimate of *a* and *B*′. Alternatively, when the extinction coefficient *a* is known (because already measured), fitting data of uncoated samples with Equation (6) gives the best estimate of the effective thermal diffusivity *α*, again together with *B*′. Both thermal diffusivity and extinction coefficient cannot be evaluated at the same time using only Equation (6), because they result strongly correlated, that is their correlation coefficient in the covariance matrix of the unknowns [17] is near to 1 and multiple couples of the two parameters are equally possible. Other authors [12,13] find that these two parameters are uncorrelated, but their data refer to highly transparent materials, glasses or similar: In such a case, the temperature trend is meaningfully different from present data, i.e., the initial temperature on the measured side is higher than the ambient one, due to transmittance.

**Figure 3:**
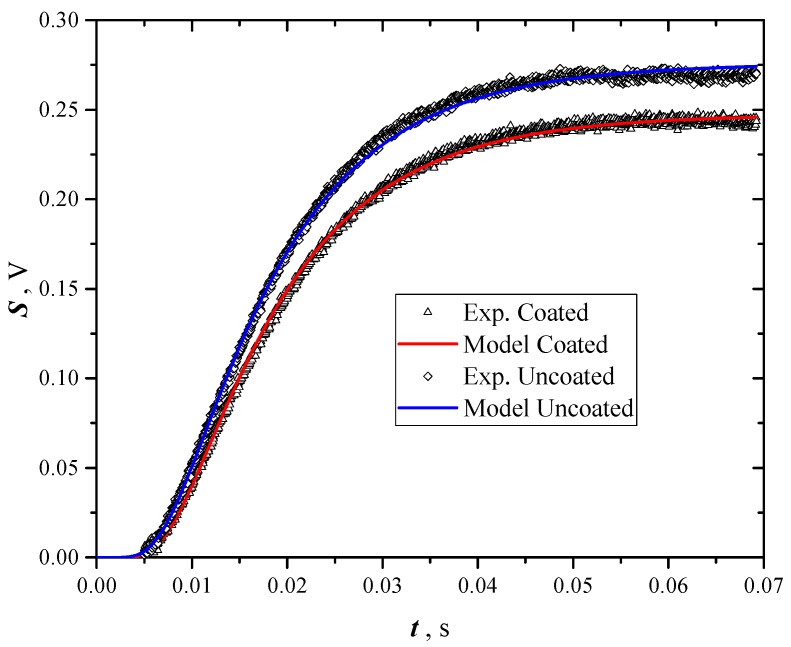
Overlapping of a typical signal recorded in a test by flash method on *GNP* coated and uncoated samples.

The procedures to find out the best estimate of the thermal diffusivity uses both data of coated and uncoated samples. First with a nonlinear regression procedure, which supplies the best estimate of *A*′ and *α* fitting data of coated samples using only Equation (3). Then the best estimate of *B*′ and *a* is given from the regression of uncoated sample data with Equation (6). Figure 3 shows a typical result of this procedure, where data of coated and uncoated samples and fitted curves are presented. Both data are used to evaluate at the same time the effective thermal diffusivity and extinction coefficient using the Equations (3) and (6) as regression models.

**Figure 4:**
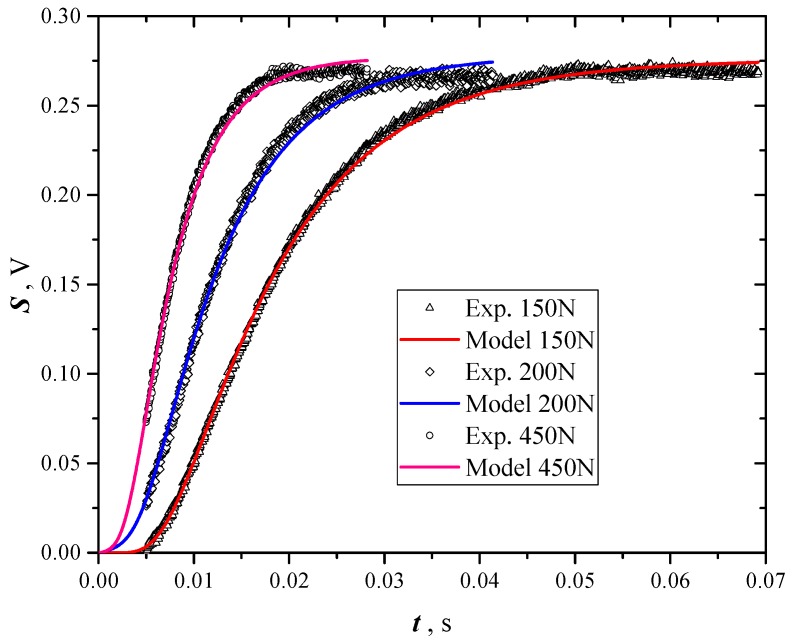
Signal recorded for three different samples of *GNP* not coated, pressed with different loads (100, 200 and 450 N).

Figure 4 shows the results of three different samples. Data of the first 5 ms are missing due to the reasons exposed in [6] (overlapping of the direct flash signal due to ambient reflections).

Driving *a* to infinite (opaque samples), Equation (6) tends to Equation (3), i.e., the thermal trend is only function of time. 

## 3. Sample Preparation and Test Setup

The conglomerate was prepared processing a dispersion of *GNP* in isopropyl alcohol by means of an ultrasonic probe for 10 min. The obtained dispersion is slowly filtered on a membrane filter of *PTFE* (pore size 0.2 µm) that allows to obtain a solid *GNP* conglomerate. Thus, these conglomerates result soft and porous, and are subsequently dried in an oven at 80 °C to eliminate the alcohol residuals. Afterward, in order to get samples with different densities, the *GNPs* were compressed at different pressures [6].

**Figure 5:**
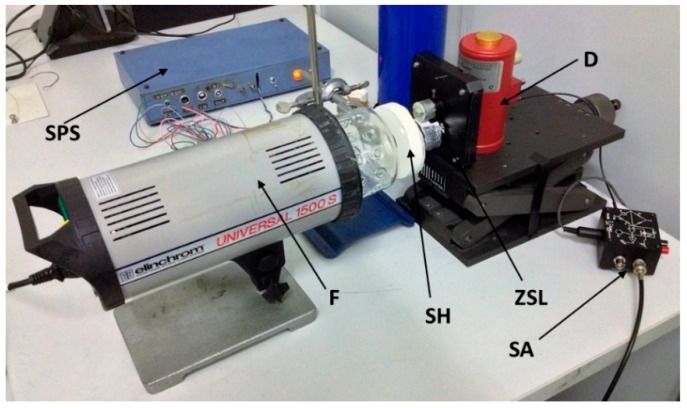
Experimental apparatus used for thermal diffusivity measurements: Flash (*F*), dewar (*D*), sample holder (*SH*), sensor amplifier (*SA*), sensor power supply (*SPS*), zinc selenide lenses (*ZSL*).

The following devices were assembled for the flash method experimental set-up (Figure 5):

- a quantum radiation detector (mercury cadmium telluride, *MCT*, active area 1 mm^2^, Pro-Lite Technology Ltd, Melton Mowbray, Leicestershire, UK), cooled with liquid nitrogen (77 K) in a dewar surrounding it;
- a ZnSe infrared lens (focal length 50 mm, manufacturer, city, state, country), transparent to visible and *IR* radiation from 0.5 to 13 µm, located in front of the *MCT* detector (*ZSL*);
- a photographic flash Universal 1500 S Elinchrom (*F*), 200 W nominal maximum power (Elinchrom SA, Renens, Switzerland). Tests were carried out with one half of this maximum power;
- data acquisition system (NI USB-6229, National Instruments, Austin, Texas, USA) set at a sampling rate of 25 kHz, and ±10 V range.

*GNP* samples obtained by filtration are relatively soft and present very low densities. Their inconsistency and brittleness prevent their handling for direct measurement. So, they were pressed with an *MTS* loading machine equipped with a load cell MTS 661.20F-02 (with applicable loads from 50 to 10,000 N, MTS Systems Corporation, Eden Prairie, Minnesota, USA). Eight samples were pressed with different loads and then coated with Aquadag^®^ on both sides. As already said, the coating thickness is negligible and was not taken into account in data processing. Table 1 shows the different loads and the resulting densities. Density was calculated measuring the sample weight (with an analytical balance with a 1 mg resolution) and its geometrical sizes.

The detected signal during tests is related to the temperature of the rear surface of the sample. In order to find out this relation, a type K flat thermocouple (homemade) was applied to the measured surface of the sample during one specific test, and an asymptotic temperature increase of 22 °C was measured, corresponding to a pyrometer signal increase of 0.226 V. Thus, a sensitivity of the pyrometer of 10.3·mV⋅°C^−1^ is deduced. The linearity between the detector output (in V) and the temperature increase (in °C) leads to write, as in [6]:

(7) S[V]=c+d·t [°C]

Through the conversion factor between signal and temperature it is possible to calculate the asymptotic temperature reached in each test. 

The applied load could change the structure of the GNP and the mutual orientation of GNP into the sample, just as discussed in [6].

In order to exclude damage in the GNP internal structure, a SEM characterization was carried out on two samples, loaded at 550 and 5000 N.

**Figure 6:**
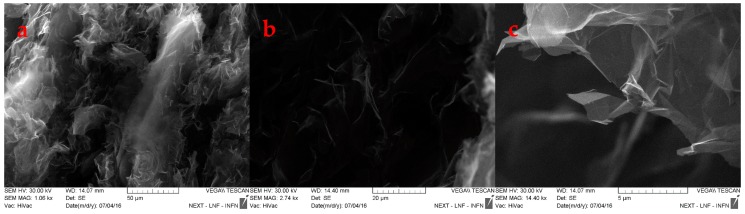
SEM images of sample 4 at different magnifications.

**Figure 7:**
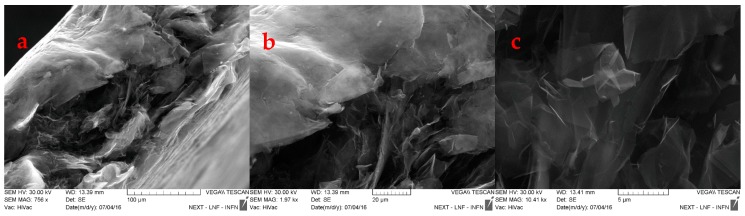
SEM images of sample 7 at different magnifications.

The SEM characterization provides some hints about the evolution of GNP samples: The images at lower magnification, Figure 6a and Figure 7a, suggest a reduction of the interparticle distances, upon increasing the loading. Nevertheless, the single particle structure is not affected by any kind of breaking: The observation at highest magnification shows more in details the modification on the interparticle distances, in which the “packaging” increases by increasing the applied load, Figure 6b and Figure 7b, but the packaging does not affect the structure of GNPs, that preserve their 2D structure, e.g., fewer number of layers and literal size grain dimension, see Figure 6c and Figure 7c.

## 4. Results

### 4.1. Thermal Diffusivity

Thermal diffusivity values are obtained with the following procedure, valid when samples are partially transparent:

- pressing samples at predefined loads;
- testing uncoated samples five times each, and evaluating the thermal diffusivity with *NL-LSF* analysis using Equation (3) as model;
- coating samples and testing them again five times, evaluating their thermal diffusivity again using *NL-LSF* with Equation (3).

**Figure 8:**
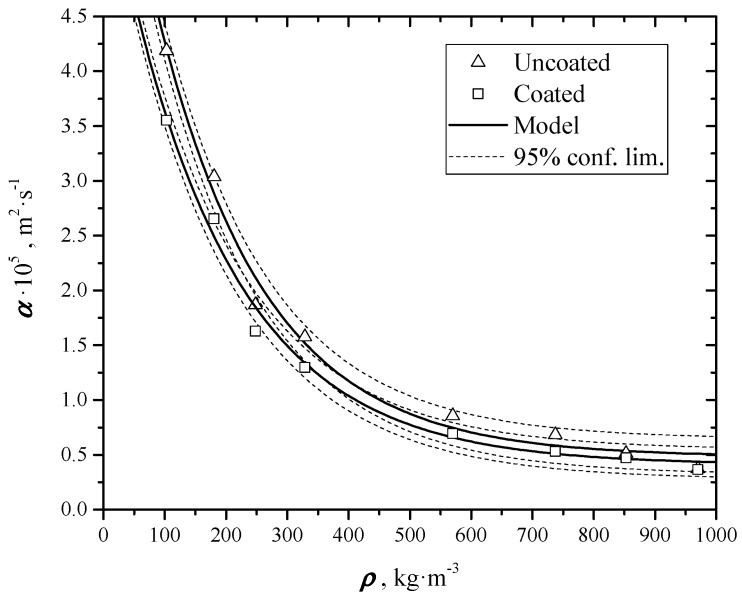
Overlapped trends of α values of the colloidal graphite coated samples and uncoated, as a function of density.

Table 2 reports these thermal diffusivity values of coated and uncoated samples obtained processing data with the same Equation (3). At the same low density, thermal diffusivities of uncoated samples result apparently higher of about 10%, while this differences tend to zero when density increases. As already said in Section 2.3, this is due to the effect of transmittance: If samples are partially transparent, the thermal wave reaches the rear surface in a shorter time as respect to opaque samples. At higher densities no difference between coated and uncoated samples is evident, being the samples are practically opaque. Figure 8 shows the experimental results, fitting curves and confidence limits for the coated and uncoated samples. For densities above 250 kg⋅m^−3^, the uncertainty bands are overlapped, that is the transmittance effects are negligible.

### 4.2. Extinction Coefficient Results

**Figure 9:**
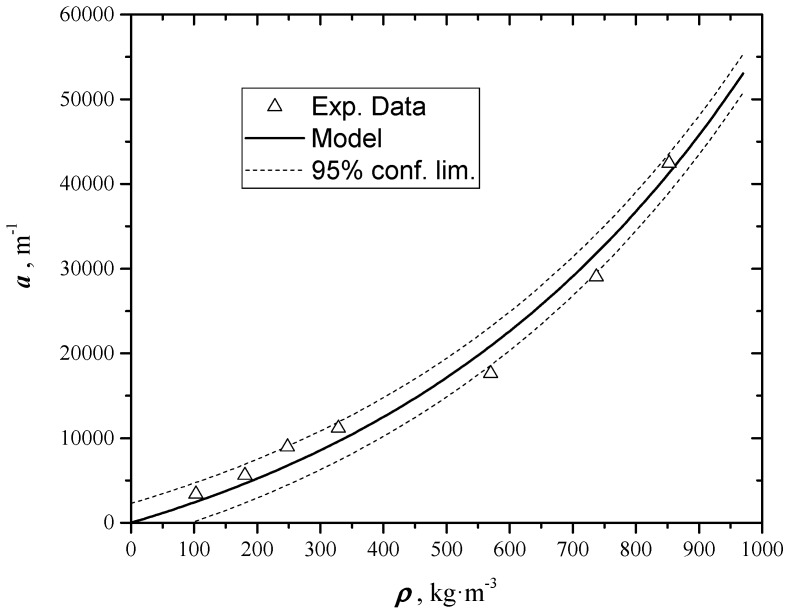
Extinction Coefficient at different density.

These results are obtained through a *NL-LSF* on data of uncoated samples, using Equation (6) as regression model. The regression parameters to be evaluated are *B*′, proportional to flash intensity, and the extinction coefficient *a*. During tests thermal diffusivity is assumed constant, using the same values already found for coated samples. In Table 3 and in Figure 9 the determined values of extinction coefficient at different densities are reported. It is evident that the extinction coefficient depends on density: In fact, as density increases porosity decreases and samples become more and more opaque. The trend of the extinction coefficient results an increasing exponential, and can be described by the following equation:

(8) $$a(\rho )=b_{1}\cdot \left[\exp(b_{2}\rho )-1\right]$$

Equation (8) is obtained assuming *a* = 0 when *ρ* = 0, and *a* = ∞ when *ρ* = ∞. A *NL-LSF* gives the best estimate of *b*_1_ (13250 ± 120 m^−1^) and *b*_2_ (1.67⋅10^−3^ ± 0.01⋅10^−3^ m^3^⋅kg^−1^).

### 4.3. Reflectance

After a transient, all samples reach the same temperature (evaluable from the asymptote of the signal and the signal/temperature conversion factor). This temperature increase is related to the absorbed heat through the following usual equation:

(9) $$Q=mc_{p}\Delta T$$

where *c*_p_ is the specific heat, *m* the sample mass, and Δ*T* the asymptotic temperature increase. As all measurements were carried out with the same flash power, all samples with equal mass and *c*_p_ should reach the same asymptotic temperature. Table 4 and Figure 7 show instead a linearly decreasing asymptotic temperature of uncoated samples, while the one of coated samples remains constant. The coating makes the surface perfectly absorbing due to the high emissivity of the Aquadag^®^ (about 0.99). So, the value of the *GNP* coated samples can be assumed as reference, because their reflectance is considered negligible. Reflectance can be calculated from the ratio of reflexed light over the incident one. Indicating with $T^{\infty }{}_{\mathrm{coat}}$ the asymptotic temperature of the coated samples and $T^{\infty }{}_{\mathrm{unc}}$ the one of the uncoated, the reflectance results:

(10) $$\beta =\frac{T^{\infty }{}_{\mathrm{coat}}-T^{\infty }{}_{\mathrm{unc}}}{T^{\infty }{}_{\mathrm{coat}}}$$

**Figure 10:**
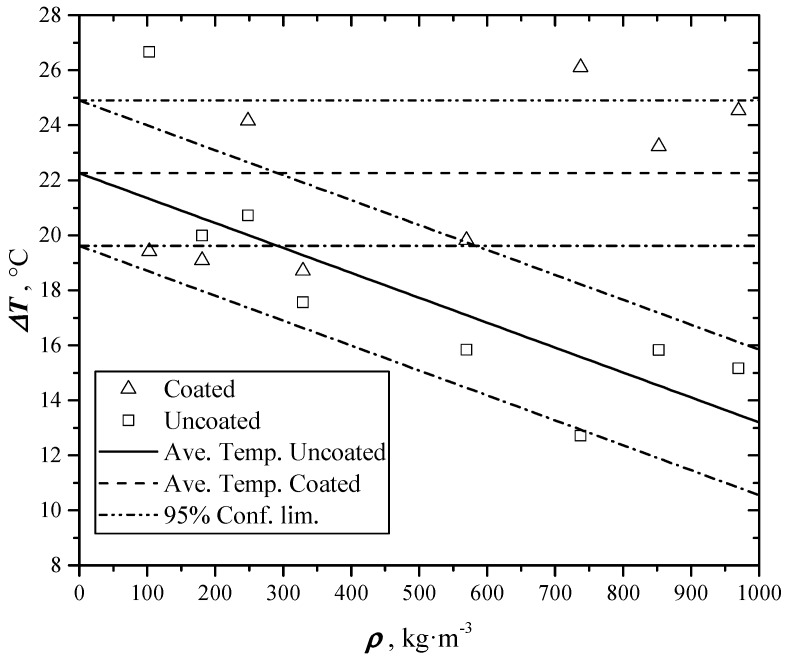
Asymptotic temperature increases during thermal diffusivity tests, for coated and uncoated samples, as a function of density.

Evaluated reflectance values from the tests are reported in Table 4 and Figure 10. The high reflectance (more than 40%) of the samples pressed with higher loads makes their surface smoother and brighter.

## 5. Extinction Coefficient Uncertainty Analysis

### 5.1. Uncertainty due to Thermal Diffusivity and Sample Thickness

**Figure 11:**
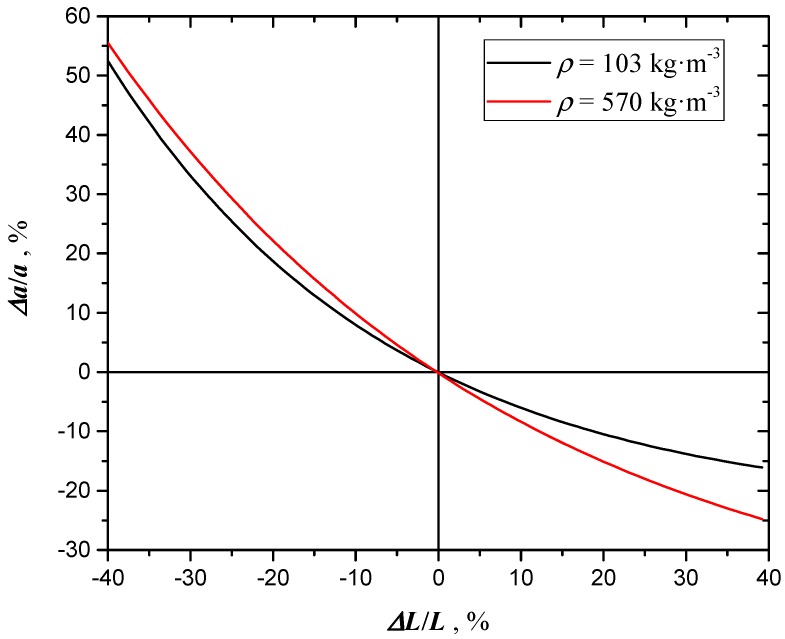
Relative deviation of *a* as a function of the relative deviation of sample thickness (at *ρ* = 570 kg⋅m^−3^).

**Figure 12:**
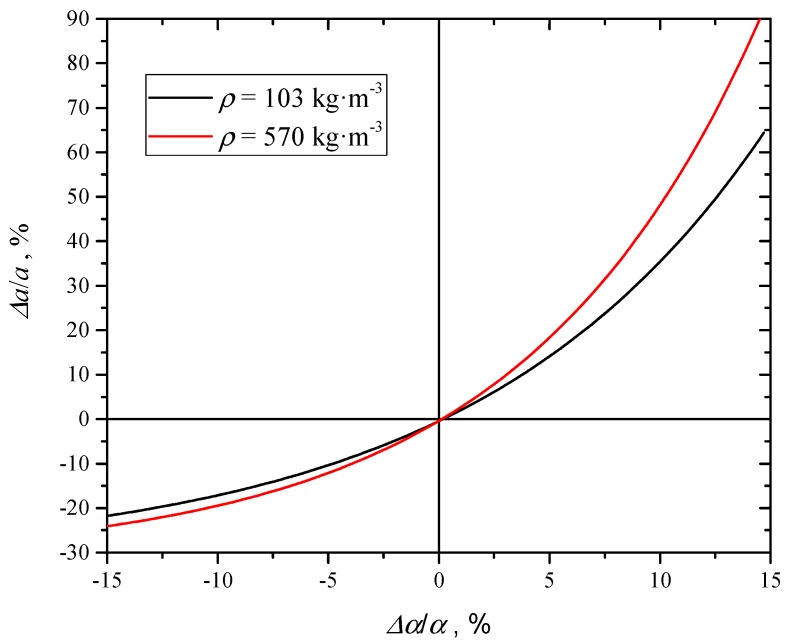
Relative deviation of *a* as a function of relative deviation of the thermal diffusivity of coated samples, at two different densities.

The extinction coefficient has been evaluated with *NL-LSF* analysis using Equation (6) as regression model. At each density, sample thickness and thermal diffusivity of coated samples were fixed. The uncertainty analysis for thickness and thermal diffusivity measurements was already carried out in [6]. In the present section the type B uncertainties (according to ISO-GUM [18]) of extinction coefficient due to the propagation of thermal diffusivity and thickness uncertainties are reported. Using previously reported data, Figure 11 and Figure 12 report the relative change of the extinction coefficient *a* of uncoated samples as a function of the relative thickness and thermal diffusivity, at constant density.

From these figures, it is apparent that the uncertainty propagates asymmetrically. Besides, Figure 12 reports two trends at different densities: It is evident that the relative deviation of the extinction coefficient at the same α increases with density.

### 5.2. Uncertainty due to Convection/Radiation Effects

**Figure 13:**
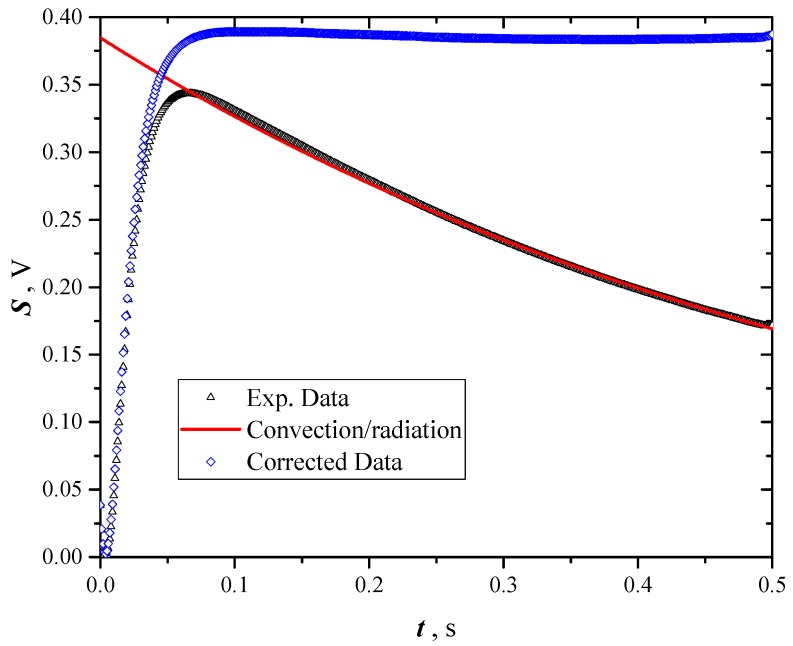
How raw acquired temperature data are corrected to take into account the convection heat transfer; red line: Pure convection trend evaluated with the lumped parameter model.

When samples are in air and the temperature of sample surface significantly increases, the hypothesis of adiabaticity can be no more valid, due to start of free convection and radiation from the sample surfaces to the ambient. In fact, the following Figure 13 show that after some tenths of a second the sample temperature begins to decrease due to the convection/radiation start. This item was already handled by other authors [10] and the same authors [19]. The algorithm used to correct data for taking into account convection/radiation start is the following:

- data after the inflection point of the whole trend (including the temperature decrease) are processed with a nonlinear least square regression using the lumped parameter solution as model:
  (11) $$T(\tau )=T_{\infty }+\left(T_{0}-T_{\infty }\right)e^{-\chi \cdot \tau }$$
  where $T_{0}$ and $T_{\infty }$ are the initial and asymptotic temperatures of the considered time range, and χ a constant function of the convection and radiation heat transfer coefficients, the thermal capacity of the sample and the area exposed to the fluid (air);
- exponential decreasing data are extrapolated till to the start of the pulse heating;
- data used for thermal diffusivity calculation are corrected adding the difference between Equation (6) and its extrapolated initial value. This procedure returns data not influenced by convection/radiation, in fact their asymptote results horizontal.

This procedure was carried out on the data reported in the present paper and compared to the original data, simply interrupted at the maximum value. The difference resulted about 3%, so the experimental data were corrected in the way above described. The small value of this correction is due to the short time of the pulse during the thermal diffusivity measurements (0.06 s), which is much less than the heat transfer due to convection/radiation (2 ÷ 3 s).

## 6. Conclusions

A homemade flash method for measuring thermal diffusivity of solid samples was applied to test graphene nano-platelets (*GNP*) in the shape of thin plates. The usual hypotheses generally assumed in the flash method (pulse heat source on the sample irradiated surface, approximated by a Dirac delta function in time, and opaque samples, i.e., a Dirac delta function also in space) were removed due to not negligible flash time length and partial penetration of light into the samples. When samples had been previously blackened with a thin layer (~1 μm) of colloidal graphite, they result completely opaque, and flash method appears to give consistent thermal diffusivity results. A data processing algorithm which compares the results of opaque and partially transparent samples was developed to evaluate the sample transmittance to the impinging flash light. The trend of the extinction coefficient versus density results is exponentially increasing.

## Figures and Tables

**Table 1:** Different tested samples, pressing load, and resulting density.

Sample #	Load (N)	Diameter (mm)	Thickness (mm)	Mass (g)	*ρ* (kg⋅m^−3^)
1	100	31	2.36	0.184	103.1 ± 1.7
2	200	31	1.49	0.201	180.7 ± 3.1
3	450	34	0.88	0.200	248.1 ± 4.8
4	550	32	0.65	0.186	352.9 ± 9.3
5	1500	34	0.39	0.200	569.3 ± 9.7
6	3000	34	0.29	0.201	785 ± 27
7	5000	34	0.23	0.184	859 ± 38
8	7000	35	0.21	0.184	950 ± 28

**Table 2:** Results of thermal diffusivity measurements of graphene samples with and without coating (* Uncoated, ** Coated).

Sample #	Load (N)	*ρ* (kg⋅m^−3^)	*α*·10^7^ (m^2^·s^−1^) *	*s_α_/α* (%)	*α*·10^7^ (m^2^·s^−1^) **	*s_α_/α* (%)
1	100	103.1 ± 1.7	419 ± 2.1	0.5	355 ± 10.3	2.9
2	200	180.7 ± 3.1	304 ± 1.2	0.4	265 ± 2.6	1.0
3	450	248.1 ± 4.8	187 ± 1.6	0.9	163 ± 1.5	0.9
4	550	352.9 ± 9.3	158 ± 0.8	0.5	130 ± 1.4	1.1
5	1500	569.3 ± 9.7	85.6 ± 1.8	2.1	69.1 ± 0.8	1.1
6	3000	785 ± 27	68.3 ± 3.7	5.4	53.0 ± 0.2	0.4
7	5000	859 ± 38	51.1 ± 0.6	1.1	47.2 ± 0.4	0.9
8	7000	950 ± 28	37.1 ± 0.6	1.6	36.6 ± 0.5	1.4

**Table 3:** Results of extinction coefficient at different densities (* Uncoated, ** Coated).

*ρ* (kg⋅m^−3^)	L (mm)	*α*·10^7^ (m^2^⋅s^−1^) *	*α*·10^7^ (m^2^⋅s^−1^) **	*a* (m^−1^)
103.1	2.36	419	355	3406
180.7	1.49	304	265	5607
248.1	0.88	187	163	8975
352.9	0.65	158	130	11,170
569.3	0.39	85.6	69.1	17,629
785	0.29	68.3	53.0	29,034
859	0.23	51.1	47.2	42,451

**Table 4:** Results of reflectance *β* at different densities (* Uncoated, ** Coated).

*ρ* (kg⋅m^−3^)	*T* (°C) *	*T* (°C) **	*β*
103.1	26.7	19.4	0
180.7	20	19.1	0.02
248.1	20.7	24.2	0.05
352.9	17.6	18.7	0.09
569.3	15.8	19.8	0.21
785	12.7	26.1	0.29
859	15.8	23.2	0.35
950	15.15	24.5	0.41

## Nomenclature

**Latin**		
*A*	proportional factor on light intensity in Equation (2)	[V]
*a*	extinction coefficient	[m^−1^]
*B*	proportional factor on light intensity in Equation (5)	[V]
*b*	nonlinear least square regression coefficient	
*c*	constant in Equation (7)	[V]
*c*_p_	specific heat capacity	[J·kg^−1^·K^−1^]
*d*	constant in Equation (7)	[V·°C^−1^]
*I*	light intensity	[W·m^−2^]
*k*	thermal conductivity	[W·m^−1^·K^−1^]
*L*	sample thickness	[m]
*m*	mass	[kg]
*Q*	thermal energy	[J]
$\dot{q}$	specific thermal flux	[W·m^−1]^
*R*	inverse of the time constant	[s^−1^]
*S*	signal	[V]
*s*	standard uncertainty (estimated)	
*T*	temperature	[°C or K]
*t*	temperature	[°C]
*x*	abscissa along the sample thickness	[m]
**Greek and composite symbols**		
*α*	thermal diffusivity	[m^2^·s^−1^]
*β*	reflectance	
*δ*	Dirac delta function	
*ρ*	density	[kg·m^−3^]
*τ*	time	[s]
χ	constant function of Equation (11)	[s^−1^]
**Subscript**		
0	start, initial value	
∞	infinity, asymptotic value	
coat	coated	
unc	uncoated	
**Acronyms**		
*D*	Dewar	
*F*	Flash	
*GNP*	Graphene Nano-Plates	
*MCT*	Mercury Cadmium Telluride detector	
*NL-LSF*	Nonlinear least square fitting	
*PTFE*	Polytetrafluoroethylene	
*SA*	Sensor Amplifier	
*SH*	Sample holder	
*SPS*	Sensor Power Supply	
*ZSL*	Zinc Selenide Lenses	

## Appendix A. Analytical Expression of the Propagation of a Double Exponential Pulse in a Solid Slab by Conduction

According to the Green function method, Equation (1) is the solution when heat generated in the slab has the shape of a Dirac delta function in time at the irradiated surface (*x* = 0).

When the effective heat is not described by this function, but by the following:

(A1) $$\dot{q}\left(x,\tau \right)=B\cdot a\cdot \exp\left(-ax\right)\left(\exp\left(-R_{1}\tau \right)-\exp\left(-R_{2}\tau \right)\right)$$

the solution can be obtained considering the overlapping of an infinite number of Dirac delta solutions with an amplitude corresponding to the intensity of the real input. I.e. the solution is the integral of Equation (1) delayed in time and in space *θ*·(*x* − *x*′, *τ* − *τ*′) multiplied by the values of Equation (A1):

(A2) $$T\left(x,\tau \right)=\int_{0}^{\tau }\int_{0}^{x}\dot{q}\left(x^{\prime },\tau^{\prime }\right)\vartheta \left(x-x^{\prime },\tau -\tau^{\prime }\right)d\tau^{\prime }dx^{\prime }$$

That is the following integral must be solved:

(A3) $$\begin{array}{l} T\left(x,\tau \right)=\int_{0}^{\tau }\int_{0}^{x}B\cdot a\cdot \exp\left(-ax^{\prime }\right)\left(\exp\left(-R_{1}\tau^{\prime }\right)-\exp\left(-R_{2}\tau^{\prime }\right)\right)\cdot \\ \cdot \left[1+2\sum_{n=1}^{\infty }\cos\left(\frac{n\pi \left(x-x^{\prime }\right)}{L}\right)\exp\left(-\frac{n^{2}\pi^{2}\alpha (\tau -\tau^{\prime })}{L^{2}}\right)\right]d\tau^{\prime }dx^{\prime } \end{array}$$

The integration variables can be separated. The time integration gives the Equation (3), already obtained in [6]. The new integral to be solved is:

(A4) $$T\left(x,\tau \right)=\int_{0}^{x}B^{\prime }\cdot a\cdot \exp\left(-ax^{\prime }\right)\left[F(\tau )+2\sum_{n=1}^{\infty }G(\tau )\cos\left(\frac{n\pi \left(x-x^{\prime }\right)}{L}\right)\right]dx^{\prime }$$

where:

$$F(\tau )=\frac{1-\exp\left(-R_{1}\tau \right)}{R_{1}}-\frac{\exp\left(-R_{2}\tau \right)-1}{R_{2}}$$

and:

$$G(\tau )=\frac{\exp\left(-R_{1}\tau \right)-\exp\left(-{\left(\frac{n\pi }{L}\right)}^{2}\alpha \tau \right)}{{\left(\frac{n\pi }{L}\right)}^{2}\alpha -R_{1}}-\frac{\exp\left(-R_{2}\tau \right)-\exp\left(-{\left(\frac{n\pi }{L}\right)}^{2}\alpha \tau \right)}{{\left(\frac{n\pi }{L}\right)}^{2}\alpha -R_{2}}$$

The solution of Equation (A4) is:

(A5) $$\begin{array}{l} T(x,\tau )=B^{\prime }\{F(\tau )\left(1-\exp(-ax)\right)+2\sum_{n=1}^{\infty }[G(\tau ){\left(1+{\left(\frac{n\pi }{aL}\right)}^{2}\right)}^{-1}(-\exp(-aL)\cdot \\ \cdot \cos\left(\frac{n\pi (x-L)}{L}\right)+\cos\left(\frac{n\pi (x)}{L}\right)-\frac{n\pi }{aL}\exp\left(-aL\right)\sin\left(\frac{n\pi (x-L)}{L}\right)+\frac{n\pi }{aL}\sin\left(\frac{n\pi (x)}{L}\right)]\} \end{array}$$

Thus the temperature response in *x* = *L* is given by:

(A6) $$T(L,\tau )=B^{\prime }\left\{F(\tau )\left(1-\exp(-aL)\right)+2\sum_{n=1}^{\infty }\left[G(\tau ){\left(1+{\left(\frac{n\pi }{aL}\right)}^{2}\right)}^{-1}\left(-\exp(-aL)+{\left(-1\right)}^{n}\right)\right]\right\}$$
